# Differences in the Development of Adverse Infusion Reactions to Rituximab in Patients With Systemic Lupus Erythematosus, Rheumatoid Arthritis and Non-Hodgkin's Lymphoma-Enigma Variations

**DOI:** 10.3389/fmed.2022.882891

**Published:** 2022-05-16

**Authors:** Sergio Gilaberte Reyzabal, David Isenberg

**Affiliations:** ^1^Internal Medicine Department, Hospital Universitario de Guadalajara, Guadalajara, Spain; ^2^Division of Medicine, Centre for Rheumatology, University College London, London, United Kingdom

**Keywords:** systemic lupus erythematosus, rheumatoid arthritis, B-cell depletion, B-cell malignances, allergic reaction

## Abstract

It has become clear that rituximab treatment is useful for both B-cell malignancies and autoimmune rheumatic diseases. However this treatment is associated with an increased risk of an allergic reaction. We have reviewed the frequency with which these reactions occur in these different conditions. They appear to be less frequent when rituximab is used to treat rheumatoid arthritis and systemic lupus perhaps because concomitant steroids are invariably given to these patients with the rituximab which is not necessarily the case with the treatment of B-cell malignancies.

## Introduction

The introduction of B-cell depletion using rituximab for the treatment of non-Hodgkin's lymphoma (1) in the late-1990s was extended within 5 years, to the treatment of both rheumatoid arthritis (RA) (2) and systemic lupus erythematosus (SLE) (3). While this approach remains very useful, in both of these autoimmune rheumatic diseases, it has become clear that the rates of infusion reactions vary in patients with different diseases.

## How Common are Infusion Reactions and What is Their Severity in Different Disorders?

### What Are the Mechanisms of Hypersensitivity Reactions Secondary to Rituximab?

Although rituximab is usually well-tolerated, its use has been linked to hypersensitivity reactions which have been classified as infusion-related, cytokine release, type I (IgE), mixed, type III and type IV reactions. Immediate infusion-related rituximab reactions are relatively common and seem to decrease in frequency with later infusions. There has been much interest in the role of circulating pre-existing or newly-synthesized human anti-chimeric antibodies (HACA) to rituximab. It has been noted (4, 5) that *in vitro* rituximab-specific IgE and Th2 cells have been identified in a patient with rheumatoid arthritis who had suffered an allergic rituximab reaction. However, HACAs do not need to be of the IgE class in order to simulate a clinical hypersensitivity reaction. Thus, some cases have been reported with IgG immunoglobulins as the culprit (6).

Non-HACA related infusion reactions can be caused by a cytokine release syndrome (CRS), which is a cytokine release from B cells following RTX-mediated lysis. This may be difficult to distinguish from true hypersensitivity, but is associated with the acute release of particular cytokines e.g., tumor necrosis factor, interferon-gamma, interleukin-6 and interleukin-2 (7).

Interestingly, it has been suggested that a relatively higher proportion of males develop these reactions both in SLE (6) and in patients with both hematological and cancer diagnoses (8, 9).

### Adverse Infusion Reactions to Rituximab in Non-Hodgkin Lymphoma

A retrospective, observational, multicenter study of pharmacovigilance studied the safety of rituximab in the treatment of patients with B-cell lymphoproliferative disorders and autoimmune diseases. 374 patients received the treatment (2,864 infusions); these reactions were observed more frequently in patients with hematologic malignancies (25% in indolent non-Hodgkin lymphoma, 35.9% in chronic lymphocytic leukemia and 28.3% in high-grade non-Hodgkin lymphoma) than in patients with autoimmune disorders (9.4%). Most of the patients with an autoimmune disorder were treated with steroids and, in some cases, with immunosuppressants, and this could explain the lower incidence of these adverse reactions in this group (10).

In a Korean study, undertaken to verify the clinical features and risk factors for infusion related reactions of rituximab, analysis of only patients with B cell malignancies revealed that corticosteroid containing prophylaxis was significantly associated with a decrease in the frequency of these events, from a 42% (among patients not given prophylaxis to 8% in those who were) (11).

In general, adverse events during infusions occur more frequently in patients with B-cell malignancies (74% in B-NHL) than in those with autoimmune disorders (i.e., 34% in RA) (12).

### Frequency of Adverse Infusion Reactions to Rituximab in Rheumatoid Arthritis

In 1998, Edwards and Cambridge attempted to verify, in an open-label trial, the hypothesis that B lymphocytes may be essential to disease perpetuation in patients with RA; they suggested that B-lymphocyte depletion may be a safe and effective therapy (2). A subsequent major double-blind trial confirmed the efficacy of rituximab in RA (13).

First infusion reactions occurred in ~25% of patients with RA (10). The majority of reactions were mild to moderate with symptoms like headache, flushing, rash, hypertension and pyrexia; however, severe infusion reactions that resulted in drug withdrawal were uncommon (<1%) (14).

In 2010, an analysis of safety data (including adverse events and infections) from patients treated with rituximab in combination with methotrexate in a global clinical trial program was performed. The rates of infusion reaction in the second, third, fourth, and fifth course of rituximab were 13, 9, 9, and 3%, respectively. Thus, they concluded rituximab remained well tolerated over multiple courses. Serious adverse events (SAE) and infections remained stable over time and by treatment course (15).

### Frequency of Adverse Infusion Reactions to Rituximab in Lupus

A detailed description of adverse infusion reactions in 136 SLE patients noted that a total of 22 patients (17.6%) had 28 (5.8% of total infusions) documented clinically significant adverse infusion reactions (6). The rate in this retrospective analysis was remarkably similar to that recorded (16.4%) in a randomized, double-blind placebo-controlled phase III trial in patients with lupus nephritis treated concomitantly with mycophenolate mofetil and glucosteroids (16). In this latter study, most infusion-related events occurred following the first infusion, becoming less frequent subsequently. However, only one serious infusion-related event was noted in the rituximab-treated arm due to a HACA positive patient (grade III).

Interestingly, it has been suggested that a relatively higher proportion of males develop these reactions both in SLE (6) and in patients with both hematological and cancer diagnoses (15, 16). As indicated above, it may be that the development of HACAs is critical in determining the likelihood of developing infusion reactions to rituximab. In a study of 57 SLE patients from the group originally reported by Hennessy et al. (13), the presence of HACAs was strongly associated with the development of infusion reactions to this B-cell depleting agent (17).

### Consequences and Causes of Variations in the Numbers of Patients Recording Adverse Reactions to Rituximab in Different Diseases

Rituximab and its biosimilars are widely used in the treatment of B cell malignancies and the major autoimmune rheumatic diseases RA and SLE. It is evident that the risk of adverse events is higher in those patients with B cell malignancies. Patients with RA/SLE may be “protected” by the more frequent use of concomitant corticosteroids and immunosuppressive therapies.

Reassuringly, it is a safe drug in clinical practice since the adverse events it produces are usually mild to moderate and the discontinuation of the drug is infrequent.

## Author Contributions

Both authors listed have made a substantial, direct, and intellectual contribution to the work and approved it for publication.

## Conflict of Interest

The authors declare that the research was conducted in the absence of any commercial or financial relationships that could be construed as a potential conflict of interest.

## Publisher's Note

All claims expressed in this article are solely those of the authors and do not necessarily represent those of their affiliated organizations, or those of the publisher, the editors and the reviewers. Any product that may be evaluated in this article, or claim that may be made by its manufacturer, is not guaranteed or endorsed by the publisher.
